# Cancer in prison: barriers and enablers to diagnosis and treatment

**DOI:** 10.1016/j.eclinm.2024.102540

**Published:** 2024-04-29

**Authors:** Jo Armes, Renske Visser, Margreet Lüchtenborg, Jennie Huynh, Sue Wheatcroft, Anthony X, Alyce-Ellen Barber, Emma Plugge, Rachel M. Taylor, Rachael Maree Hunter, Elizabeth Anne Davies

**Affiliations:** a School of Health Sciences, University of Surrey, UK; b Faculty of Education, University of Oulu, Finland; c National Disease Registration Service, NHS England, UK; d Cancer Epidemiology and Cancer Services Research, Centre for Cancer, Society & Public Health, Comprehensive Cancer Centre, King's College London, UK; e Revolving Doors Agency, 90 London Rd, Elephant and Castle, London, SE1 6LN, UK; f Faculty of Medicine, University of Southampton, UK; g Centre for Nurse, Midwife and Allied Health Professional Research (CNMAR), University College London Hospitals NHS Foundation Trust, UK; h Applied Health Research, Institute of Epidemiology and Health, University College London, UK

**Keywords:** Cancer care, Prison healthcare, Qualitatative methods, Survey methods

## Abstract

**Background:**

Approximately 82,000 people are in prison annually in England and Wales. Limited research has investigated cancer in this population and none has explored experiences of imprisoned people with cancer. This study aimed to address this gap.

**Methods:**

We conducted 55 semi-structured, qualitative, audio-recorded interviews with: imprisoned people with cancer (n = 24), custodial staff (n = 6), prison healthcare staff (n = 16) and oncology specialists (n = 9). Data were collected 07/10/2019–20/03/2020. Patients were recruited by prison healthcare staff and interviews were conducted face-to-face. Professionals were recruited via professional networks and interviews were conducted face-to-face or via telephone. Transcribed interviews were analysed using reflexive thematic analysis. We also analysed relevant National Cancer Patient Experience Survey (NCPES) questions for those diagnosed in prison (n = 78) and in the general population (n = 390).

**Findings:**

Our findings highlight the complexities of cancer care for imprisoned people. We identified three core themes: control and choice, communication, and care and custody. Whilst people in prison follow a similar diagnostic pathway to those in the community, additional barriers to diagnosis exist including health literacy, the General Practitioner appointment booking system and communication between prison and oncology staff. Tensions between control and choice in prison impacted aspects of cancer care experience such as symptom management and accessing cancer information. NCPES results supported the qualitative findings and showed people in prison reported significantly poorer experiences than in the general population.

**Interpretation:**

Our findings demonstrate the complexity of cancer care in custodial settings, identifying barriers and enablers to equitable cancer care provision and offering insights on how to improve care for this population.

**Funding:**

National Institute for Health and Social Care Research Delivery Research Programme 16/52/53 and Strategic Priorities Fund 2019/20 10.13039/501100013589Research England via University of Surrey.

**Research in context**

**Evidence before this study**

Previous research on cancer in prison populations has focused on screening and the prevalence and incidence of specific cancer types. Results highlight that people with cancer in prison often have poorer health outcomes compared to the general population and often have multiple and complicated problems, yet little is known about their experiences of being diagnosed and treated for cancer whilst in prison.

**Added value of this study**

This study adds to existing evidence by outlining pathways to diagnosis, challenges of treatment and follow-up care, and patient and professional experiences of cancer care when a patient resides in prison. Importantly it provides insights into the specific complexities of providing cancer care in a prison environment.

**Implications of all the available evidence**

Structural barriers mean that receiving a diagnosis of cancer is more difficult for people in prison. They have limited access to information about their illness and have to manage symptoms and side-effects in a challenging environment with limited emotional and social support. This has significant implications for the diagnosis and management of cancer care in prison and how these patients are managed in secondary care.

## Introduction

People in prison worldwide often have multiple and complicated healthcare needs.^1^^,^^2^ Research on the prison population to date has tended to focus on communicable diseases, mental health and a small number of non-communicable diseases^3^ and results show outcomes tend to be poorer than for the general population. People in prison should receive equivalent healthcare to people in the community.^4^ However, recent research on secondary care use by people in English/Welsh prisons showed multiple factors that potentially hinder people from receiving the same level of treatment and care as others.^5^ These include security overriding healthcare needs, delayed access to healthcare due to prison regimes and restricted autonomy limiting self-management of health.^5^

While it is known that cancer affects people in prison, studies have focused on prevalence and incidence rates^6^, ^7^, ^8^ at a local or regional level and/or on cancer screening uptake.^9^^,^^10^ Little is known about the process of diagnosis or the type and level of care people in prison receive during cancer treatment. Likewise, the impact of cancer on people in prison and professionals working with this group is unclear. Since 2013, prison health care is primarily the responsibility of NHS England, which directly commissions primary, hospital and public health services for people in prison.

Our mixed methods study aimed to address this evidence gap by investigating the incidence, clinical outcomes, cost of care and experience of people diagnosed with cancer in English prisons. An epidemiological study investigated cancer incidence, curative cancer treatment and survival rates^11^ and a health economic study compared health care costs.^12^ This paper explores experiences of being diagnosed and treated for cancer whilst in prison from the perspective of patients and professionals.

## Methods

We adopted an exploratory sequential mixed methods approach comprised of two elements: qualitative exploration of experiences of diagnosis and care of people with cancer in prison; and analysis of National Cancer Patient Experience Survey (NCPES) data. In this design the quantitative data is secondary and supplements the qualitative data. We collaborated with three Experts by Experience (EbE) (A-EB, SW, AX), all with lived experience of prison who, acting as lived experience researchers, helped design the study and conduct the interviews. EbE involvement in the study was supported by Revolving Doors (RD) and a reflection on this collaboration published.^13^

### Qualitative interviews

We undertook semi-structured, audio-recorded interviews with people in prison with experience of cancer and NHS prison health professionals, prison officers and NHS cancer clinicians who had experience of caring for people in prison with cancer. For each participant group separate interview guides and information sheets were developed. Experts from each group commented on the interview guides, to ensure all relevant topics were covered. Our EbE were involved in the design of the information sheet and interview guide to ensure information was presented in the right tone and literacy level.

The study received research ethics approval from Health Research Authority (REC 19/LO/1073) and Her Majesty's Prison and Probation Service (Ref: 2019-306).

### Sample and setting

We employed purposive sampling to recruit a sample reflecting the current prison population by gender, age, region and prison security category. Therefore, we aimed to recruit fewer women as most (96%) people in prison are men.^3^ People with cancer in English prisons were eligible to participate if they were >18 years, been diagnosed with cancer while in prison or were receiving treatment for cancer while in prison, could speak and understand English, and were judged able to provide informed consent by local prison staff. Prison healthcare staff provided potential participants with a copy of the information sheet and a verbal outline of the study aims and what it involved, gained verbal consent and arranged the date and timing of interviews with participants and RV. Understanding of the study aims and methods for data collection were checked by the researcher prior to participants signing the consent form, which allowed gender self-assignment.

Professional participants were recruited via snowball invitations, email lists of relevant professional organisations, newsletters and social media. Participants were eligible if they had experience of working with people diagnosed with and treated for cancer whilst in prison.

### Data collection

Most of the interviews were audio-recorded and transcribed verbatim by a professional transcription service. On one occasion the recording device was not permitted in the prison and so the researcher (RV) took notes. RV wrote field notes after each interview. All interviews except one were conducted in English; one patient interview was conducted in Dutch (by RV, a native Dutch speaker, who transcribed the interview in Dutch and then translated it into English). All participants provided written informed consent prior to participating.

All interviews with people in prison were conducted by RV face-to-face either alone (n = 12), or with an EbE (SW or AX) (n = 11) or researcher (JA) (n = 1).^13^ Interviews lasted, on average, 60 min and were conducted in a separate room in the prison healthcare facility. No prison staff were present during interviews. All interviewers were female, except one EbE who was male, and were unknown to participants. Both RV and JA are experienced in undertaking qualitative interviews. RV and JA provided training (6 h) to the EbE who co-conducted interviews and this included a discussion on interest in the topic and potential biases each may bring.^13^ Training was augmented by debrief and mentorship after each interview.

Interviews with prison and oncology professionals were conducted by RV either face to face or via telephone, who was unknown to them, lasting approximately 30 min.

### Role of funding

The funder of the study had no role in study design, data collection, data analysis, data interpretation or writing of the report. JA & RV had access to the data. All authors decided to publish the study findings.

### Analysis

#### Qualitative interview data

We used reflexive thematic analysis^14^ as this supported an experiential orientation whereby precedence is given to the meaning ascribed by the participant. Initially RV and JA inductively coded the interviews. Subsequently our EbE (SW, AX, AB) reviewed transcripts and identified additional codes. Coding continued in NVivo 12 and then we reviewed codes to develop and refine them into themes and subthemes. Analysis of each participant group was undertaken separately and themes and sub-themes subsequently compared across groups. All analyses were reviewed by the wider research team and our EbE and any refinements made.

All quotations are anonymised to protect the identity of participants and organisations, and participants given an identity number throughout.

#### National cancer patient experience survey

NCPES data were used to quantitatively assess experiences of cancer care between patients diagnosed with cancer in prison and in the general population (details in supplementary file 1). NCPES contains around 70 questions covering different aspects of care.^15^ For the analysis included here, NCPES data from 2012 to 2018 were used. Once the qualitative analysis was completed questions that related to the themes developed from the interviews were included in the analysis. People with a cancer diagnosed in prison were identified from the national cancer registration data based on postcode of residence at diagnosis. Linkage with NCPES is based on patient and tumour identifiers. A matched cohort approach was used to compare experiences of people diagnosed in prison with those diagnosed with cancer outside. NCPES responders outside prison were matched in a 5:1 ratio on gender, age group (18–20, 21–24, 25–29, and ten-year age group thereafter up to 79, and 80+) and NCPES data year to the NCPES responders with a cancer diagnosis made in prison. Due to the limited numbers of NCPES participants cohort matching on tumour site could not be carried out, instead it was restricted to tumour sites present in the prison patients, excluding males with breast cancer. The final sample included 78 people diagnosed in prison and 390 from the general population. Answers to NCPES questions were dichotomised in line with previous analyses.^16^ Missing answers, and those indicating the question was not applicable were excluded. Logistic regression modelling was used and adjusted for the cohort matching variables.

All analyses of NCPES data were undertaken by JH and ML who worked within the National Cancer Registration and Analysis Service (NCRAS) in the National Disease Registration Service (NDRS). NCRAS data included in this study were collected and analysed under the National Disease Registries Directions 2021, made in accordance with sections 254 (1) and 254 (6) of the 2012 Health and Social Care Act.

## Results

We conducted 55 semi-structured interviews: 24 patients from six prisons, six custodial staff, 16 prison NHS health professionals and nine NHS oncology professionals. Although we intended to conduct more interviews, data collection was halted on 20th March 2020 due to the COVID-19 pandemic when prisons were closed to external visitors. Table 1 shows personal and clinical characteristics for patient participants. Most were men (n = 21), with the most common diagnosis being prostate cancer, while for women (n = 3) this was breast cancer. Table 2 shows the roles held by the professionals interviewed.

**Table 1:** Patient personal & clinical characteristics (n = 24).

	Number
**Age bands–years**	
20–39	2
40–59	9
60–79	10
>80	3
**Cancer diagnosis**	
Breast	3
Gastrointestinal	1
Haematological	5
Head & neck	4
Lung	2
Skin	1
Urological	8
**Place of diagnosis**	
Community	7
Prison	17
**Treatment received**	
Surgery	3
Systemic anti-cancer therapy	10
Radiotherapy	4
Hormone therapy	1
None	7
**Residence during treatment**	6
Community	6
Prison	18
**Treatment status at time of interview**	
On treatment	6
Follow-up	14
Palliative care	1
No treatment received	3

**Table 2:** Professional participants job title.

Job title	Setting	N
Consultant oncologist	Hospital	2
Surgeon	Hospital	1
Clinical nurse specialist	Hospital	2
Chemotherapy scheduler	Hospital	1
Radiographer	Hospital	1
GP	Prison	5
Prison officer	Prison	4
Specialist nurse	Prison	3
Head of healthcare	Prison	3
Clinical nurse	Prison	4
Social care	Prison	1
Custodial manager	Prison	2

**Fig. 1:**
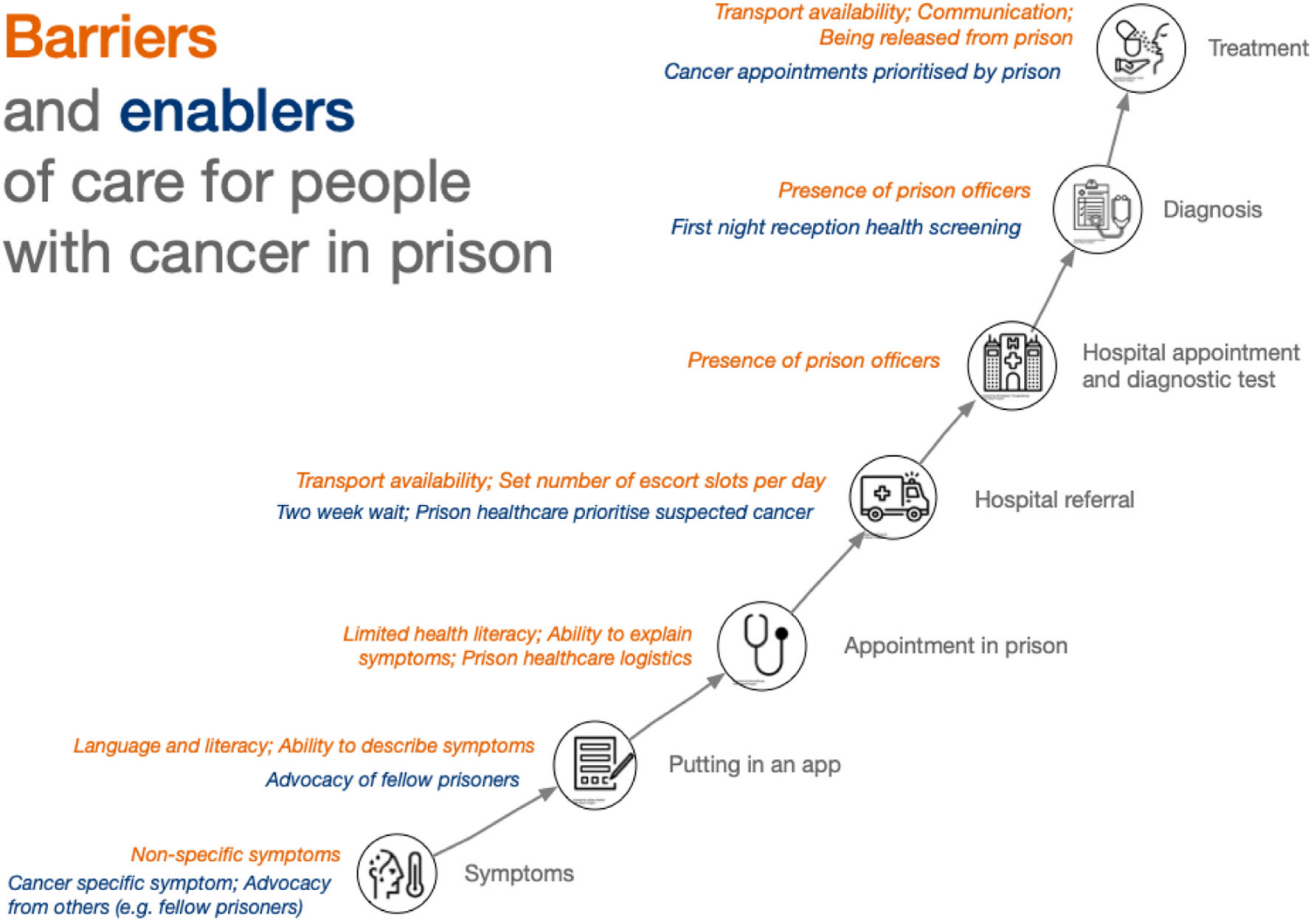
Barriers and enablers to diagnosis for people with cancer in prison. Credits for images: Treatment—Nithinan Tatah from Noun Project (CC-BY); Diagnosis—Amethyst Studoi from Noun Project (CC-BY); Hospital Appointment—Phoniaphat Thongsriphong from Noun Project (CC-BY); Hospital referral—Nawiconm from Noun Project (CC-BY); Appointment in prison—DinosoftLab from Noun Project (CC-BY); Putting in an app—Arslan Shahid from Noun Project (CC-BY); Symptoms—Noun Project (CC-BY).

In reporting our results we draw on all participant groups to highlight the particular complexities of cancer care when a patient resides in prison. The term ‘people with cancer’ is used throughout to refer to people diagnosed, treated and monitored for cancer or cancer recurrence. For each theme and sub-theme we summarise the findings, which we support by quotes presented in Supplementary file 2. We also highlight results from the NCPES analysis where they support or differ from the qualitative findings (See Table 3). Fig. 1 depicts the generic pathway to a cancer diagnosis,^16^ adapted to reflect experiences of people in prison. This pathway starts with a person experiencing symptoms, in prison they put in an application (‘app’) to get an appointment with a GP and potentially have diagnostic tests (blood tests). If cancer is suspected, they are referred to an external hospital for further diagnostic tests, receive a diagnosis and are subsequently treated for cancer. Fig. 1 also summarises the enablers and barriers at each point in the pathway which we discuss in more detail below in relation to our themes.

**Table 3:** Likelihood of preferred responses to CPES questions on cancer care among persons with a cancer diagnosed in prison compared with those diagnosed in the general population.

#	Question	Question heading	Preferred answer	Odds ratio	95% CI		p-value
1	Before you were told you needed to go to hospital about cancer, how many times did you see your GP (family doctor) about the health problem caused by cancer?	Pre-diagnosis GP visits	Saw GP no more than twice before referral to hospital	0.35	0.19	0.64	0.001
3	How long was it from the time you first thought something might be wrong with you until you first saw a hospital doctor?	Time to secondary care	Seen in <3 months	0.33	0.19	0.58	<0.0001
11	When you were first told that you had cancer, had you been told you could bring a family member or friend with you?	Family or friend on first visit	Yes	0.17	0.09	0.32	<0.0001
14	When you were told you had cancer, were you given written information about the type of cancer you had?	Written information about cancer	Yes, and it was easy to understand	0.66	0.38	1.14	0.135
17	Were the possible side effects of treatment(s) explained in a way you could understand?	Possible side effects of treatment explained	Yes, definitely	0.90	0.51	1.61	0.73
18	Before you started your treatment, were you given written information about the side effects of treatment(s)?	Written information about side effects of treatment	Yes, and it was easy to understand	0.22	0.09	0.56	0.001
26	Did hospital staff discuss with you or give you information about the impact cancer could have on your work life or education?	Information on impact on work or education	Yes	0.53	0.26	1.09	0.082
48	Were you given enough privacy when discussing your condition or treatment?	Privacy	Yes, always	0.20	0.10	0.41	<0.0001
54	Did hospital staff tell you who to contact if you were worried about your condition or treatment after you left hospital?	Contact information after leaving hospital	Yes	0.10	0.04	0.23	<0.0001
55	Did the doctors or nurses give your family or someone close to you all the information they needed to help care for you at home?	Information on care passed on to someone close	Yes, definitely	0.49	0.25	0.98	0.044
63	As far as you know, was your GP given enough information about your condition and the treatment you had at the hospital?	Enough information given to GP	Yes	0.15	0.06	0.36	<0.0001
64	Do you think the GPs and nurses at your general practice did everything they could to support you while you were having cancer treatment?	GP support	Yes, definitely	0.46	0.26	0.83	0.009

Multivariable logistic regression models, adjusted for survey year, age, gender, tumour stage and ethnicity.

### Getting a diagnosis

Cancer patients in prison are a diverse group and we identified three differing pathways to diagnosis.

1) Pathway 1: people diagnosed and treated in the community who need follow-up care in prison (n = 6).

This group were diagnosed and treated prior to going to prison but required ongoing anticancer medication, monitoring and follow up care.

2) Pathway 2: people diagnosed in the community and treated in prison (n = 1).

One participant completed all diagnostic tests shortly before being sentenced but underwent treatment in prison, highlighting the blurring of boundaries and overlaps between prison and community settings.

3) Pathway 3: people diagnosed and treated in prison (n = 17).

Pathway 3 included varied ways of being diagnosed. Some people were diagnosed via the health check that occurs during the first-night intake (‘reception screening’), whilst others were diagnosed through national screening programmes offered in prison. Some participants were asymptomatic and were diagnosed secondary to other health interventions. In contrast, some, particularly younger participants with non-specific symptoms, experienced a prolonged route to diagnosis. Oncology professionals reported that being moved between prisons could delay diagnosis. This was corroborated by a man in his 50s who was diagnosed with leukaemia via reception screening but moved to another prison where staff were unaware of this diagnosis and repeated the diagnostic process.

Three core themes were developed inductively - communication, care and custody and control and choice.

### Communication

A major barrier in providing or receiving good care is communication. This includes communication between patients and clinicians and between professional groups.

#### Between patients and GPs - getting a referral

Typically, if a person experiences symptoms in prison, they ‘put in an app [application]’ to be seen by prison healthcare. This can be paper-based or electronic but is triaged by a prison health professional who decides which patients will see a prison GP, and how quickly. Only patients and prison healthcare interviewees spoke about the ‘app’ system. Using the ‘right’ language on an application was key; one person commented that he always wrote *‘due to my history with cancer…*’ to ensure he was seen promptly. Others reported putting in daily ‘apps’ for weeks or months before receiving an appointment. Some not only filled out ‘apps’ for themselves but also helped fellow prisoners for whom literacy was a challenge. This system of ‘putting in apps’ is thus the first barrier that some people in prison needed to overcome to access healthcare.

#### ‘Malingering’ and trust

The main difficulty in detecting cancer from a prison healthcare perspective was differentiating between people with physical healthcare problems and those with mental health and substance abuse issues. Prison doctors reported that the risk of ‘*malingering*’ was high. While patients reported the challenge of ‘*not being believed’* by health professionals and custodial staff, this was not simply unwillingness on behalf of professionals but, instead, a complicated dynamic between understaffing and limited healthcare appointments, combined with a complex patient population and low cancer awareness. The implication is that people in prison may have to convince the prison GP that further investigations are warranted. In our study there were several patients in their twenties who struggled to get a diagnosis as they experienced vague symptoms and were labelled as ‘*lazy*’ or ‘*attention seeking*’.

The NCPES data corroborated these findings in that people with cancer diagnosed in prison were significantly less likely to report that ‘they saw their GP less than three times’ before being referred to hospital (odds ratio (OR) 0.32, 95% confidence interval (CI) 0.18–0.59) or that they were seen by a hospital doctor within three months (OR 0.33, 95% CI 0.19–0.59).

#### Communication between patients and cancer professionals

Oncology specialists only became involved when a patient was referred to hospital and this was the only time patients in prison could ask them questions about their cancer. Patients could not communicate directly with them from prison, as it was generally not possible to add their phone numbers to their approved numbers in a timely fashion. Thus, for patients in prison preparing questions for these visits was crucial. Oncology professionals reported offering ‘the same’ kind of information regarding reporting side-effects despite acknowledging that people with cancer in prison may struggle to contact cancer services using the telephone hotline and that this may result in poorer side effect management. It was generally accepted by patients that they had limited access to cancer specialists. Some tried to access information through Macmillan Cancer Support, a UK cancer charity. But just as patients could not easily add the number of their cancer specialists to the list of phone numbers they were permitted to call, it was equally difficult to contact charities. In some prisons Macmillan professionals visited cancer patients regularly, but this service was based on available funding thus some patients and professionals reported how Macmillan ‘*used to visit*’.

These communication challenges were reflected in the NCPES findings. People diagnosed with cancer in prison were almost as likely as those from the general population to respond that the way the possible side effects of treatment(s) were explained was easy to understand (OR 0.90, 95% CI 0.51–1.61), and that written information was much less likely to be reported as easy to understand (OR 0.22, 95% CI 0.09–0.56). Likewise, people diagnosed in prison were 90% less likely to report that they were told who to contact if they were worried about their condition or treatment after leaving the hospital (OR 0.10, 95% CI 0.04–0.23).

#### Communication between professional groups

All three professional groups reported that communication with each other was difficult. Oncology specialists reported finding it hard to communicate with prison healthcare, while prison healthcare reported that oncology services were reluctant to share information with prison healthcare. The interviews with oncology professionals revealed they found prisons mysterious places, and their narratives included many questions around what was available to patients in prison, and who to speak to. Prison officers reported being constrained by the notion of medical confidentiality, and this was highlighted by the fact that they were often not informed about a prisoner's cancer diagnosis. When prison officers tried to acquire information, prison healthcare were reluctant to share information due to medical confidentiality constraints. Prison officers noted that people in prison might inform them about their diagnosis, but as *‘malingering*’ was considered a big issue in prisons, prison officers often wanted to verify this information. Our study revealed that patients were unaware of the ways hospitals and prisons communicated about their healthcare, but they were affected by miscommunication or non-communication between oncology specialists and the prison.

Responses to NCPES showed a similar finding whereby people diagnosed with cancer in prison were far less likely to report that their GP was given enough information about their condition and the treatment received at the hospital (OR 0.15, 95% CI 0.06–0.36) than those diagnosed in the general population. Moreover, they were around half as likely to say the GPs and nurses at their general practice did everything they could to provide support during cancer treatment (OR 0.46, 95% CI 0.26–0.83). In addition, people diagnosed in prison were more likely to respond their GP was not involved (p = 0.011, data not shown).

### Control and choice

People in prison had limited control and choice about decisions regarding their own health and there were various structural barriers that hindered access to healthcare. While these barriers impact the everyday lives of people in prison the most, prison healthcare and custodial staff were also constrained by their working environment and the role divisions within prison. Oncology services by contrast aimed to empower patients to self-manage their illness, for example through exercise and healthy eating, but the prison environment provided limited opportunities for doing this.

#### Preparing for appointments

Cancer treatments are generally given at regular intervals with clear scheduling information being given to cancer patients. Because of security concerns, this information is not provided to people in prison with cancer. Patients often deduced when their next appointment was likely to be, yet oncology professionals were discouraged by prison officers from openly discussing timeframes for treatment plans. This impacted the consultation with oncology specialists. It was important people in prison were as prepared as possible to ask the right questions about side-effects, treatment, and follow-up care. If patients were not prepared, or forgot to ask questions, they missed their chance to ask any questions until the next appointment.

#### Treatment

Oncology professionals reported that treatments should not differ for patients residing in prison to those residing in the community. Yet patients reported they had less access to information about their specific cancer and, as family and friends were typically absent from the diagnostic process, they made decisions about treatment on their own and on the spot. Some participants were diagnosed prior to entering prison so their family were involved in the decision-making process. Typically, treatments such as radiotherapy and chemotherapy were only provided in hospital. Oral chemotherapy could be offered ‘in-house’, and a few participants in our study were ‘*on tablets*’ to treat their cancer.

Again, these findings are reflected in the NCPES results. People with cancer in prison were significantly less likely to report that they were told they could bring a family member or friend with them when they were first informed about their cancer (OR 0.17, 95% CI 0.09–0.32), and that family or someone close to them was given all the information they needed to help care for them at home (OR 0.49, 95% CI 0.25–0.98). Further inspection of the data not included in the modelling for the latter question (data in Supplementary Table), revealed that far more people diagnosed in prison indicated that no family or friends were involved (p < 0.0001, data not shown).

#### Managing treatment side-effects

It can be difficult to manage side-effects in prison. Oncology specialists reported the challenge of getting patients to hospital if they experienced side-effects that needed immediate medical attention. Furthermore, their accounts revealed they were not sure who was responsible for monitoring patients in prison. Patients reported a range of experiences; some did not experience any symptoms, whilst others experienced severe and ongoing side-effects. The physical prison environment was noted to be “*dirty*” which was particularly concerning for immunosuppressed patients. Prison health professionals reported there were specific protocols in place to manage side-effects. As prisons had individual responses to managing cancer care it was unclear whether these protocols were developed locally or nationally.

#### Managing emotions

Prisons are emotionally complicated places for both people in prison and for staff. Patients and prison officers reported that showing vulnerability was avoided in prison. A prison sentence is already emotionally challenging, and a cancer diagnosis adds to this. Yet, as one participant reported: *“This is not a place to have a mental breakdown*”. Patients in prison tried to save face and keep their emotions to themselves, for example when they heard their prognosis and bad news in front of prison officers. One person was hospitalised at the time of diagnosis and received his diagnosis whilst under 24-h surveillance. He reported longing to return to his cell so he could cope with this news on his own.

### Care and custody

For both prisons and healthcare organisations safety is a top priority, however the focus differs between them; for healthcare the focus is the safe delivery of care whilst for prisons the emphasis is on ensuring the safety of prisoners and protecting the public from them. Tensions that were identified between care and custody largely derived from these different foci for ensuring safety. Interviews with the professional groups showed that those working in the criminal justice system were not always sure whether their role is to provide care or custody. Prison officers found it particularly difficult to reflect on their role in the care of people in prison, despite, for example, being instrumental in getting people in prison to hospital appointments. Cancer patients in prison moved between the identity of ‘cancer patients’ and ‘prisoner’ both within the prison walls and during their out- and in-patient hospital appointments.

#### Getting to hospital

Transporting people from prison to hospital requires careful logistical planning. Each prison can convey a limited number of people (prisoners) to hospital appointments each day, based on the prison officers available for escort duty. Typically, two prison officers escort the patient and are only told on the day of duty. Our study showed that cancer was considered as urgent within prisons, and patients needing to attend for treatment or appointments were prioritised over others. Before diagnosis, people in prison are at risk of their diagnostic appointments being cancelled or replaced by others. Patients also reported missing appointments or being late when transport did not show up. Staff shortages and emergency situations within prisons could also result in missed appointments if escort officers were asked to cover other jobs within the prison.

The tension between care and custody is made visible as people in prison are handcuffed to an escorting prison officer. Prisons, however, adopted individual strategies to manage the issue of handcuffing. One prison did not require handcuffing for patients >65 years if their security risk allowed it. In another, people with a cancer were not handcuffed. This decision was based on the risk they posed to the public. For patients, not being cuffed helped minimise feelings of shame as they looked less like a prisoner. The use of handcuffs was a barrier to accessing care and was a reason for patients to refuse a hospital appointment.

#### Presence of prison officers in medical consultations

Interview data highlighted that the tension between security and autonomy for both patients, prison officers and health professionals was amplified by the presence of prison officers in medical consultations. Oncologists reported trying to ignore the prison officers, and prison officers reported various coping strategies during consultations, ranging from *“trying to absorb some information*” to “*zoning out*”. Patients had diverging opinions about the presence of prison officers, some said they did not mind it whilst others reported they would not ask certain medical questions (e.g. impact on fertility) or raise concerns in front of the officers. Reasons for this ranged from being embarrassed to being wary that officers might feedback personal information to others in the prison.

Likewise results from NCPES show those diagnosed in prison were far less likely to respond that they were always given enough privacy when discussing their condition or treatment compared with those diagnosed in the general population (OR 0.20, 95% CI 0.10–0.41).

## Discussion

This is the first study to explore experiences of cancer care in the prison population from the differing perspectives of people with cancer, prison officers, prison health professionals and oncology professionals. Our study showed that cancer care in prison was complex, not least because people in prison moved between healthcare and prison environments. People in prison followed a similar diagnostic pathway to other patients but experienced several barriers to diagnosis. Similarly, once treatment started, they were often unable to follow the advice of oncology professionals for managing and reporting any side effects. Instead, they were reliant on prison officers and health professionals to acknowledge that their symptoms were caused by the cancer treatment and respond appropriately.

A key barrier to being diagnosed was the process of “*putting in an app”*, and this was exacerbated by low literacy levels. This supports findings from other studies in the UK^5^^,^^17^ and internationally^18^ that have reported how triage and gatekeeping practices hinder equivalence of access to healthcare in prisons. Vague, non-specific symptoms, not readily recognised as reg flag indicators of cancer, are known to be a cause of prolonged diagnosis,^19^ however people in prison do not have the option to see an alternative medical practitioner if they feel their concerns are not being adequately investigated.

People in prison have the right to respect for autonomy and this includes patient confidentiality.^20^ The negative impact of prison officers in medical consultations reported in our study has been identified by other researchers,^5^^,^^17^^,^^18^ however ours is the first to highlight that oncology professionals and prison officers are also discomforted by this practice. We also present evidence that, based on protecting medical confidentiality, information needed to provide best care and support to people whilst in prison was not being shared between professional groups and at times this was reported to result in negative outcomes for people in prison. A balance between respect for autonomy and non-maleficence needs to be struck and this could be achieved by having an explicit, documented agreement between the person with cancer and both the prison and oncology professionals about what information can be shared and with who.

Oncology services frequently advise patients to bring a family member or friend to out-patient appointments to support them psychologically and help with information gathering and retention. Most of those who were diagnosed in prison reported attending appointments without any family support and little interaction between their family and the oncology team. This highlights the limited opportunities people in prison have to gain more information about their condition. This lack of family involvement was compounded by their constrained ability to communicate with family members, which our participants reported impacted negatively their emotional well-being.

Prisons are designed to take away elements of control and choice,^21^ yet hospitals aim to be inclusive and empowering. The overlap and strain of moving between the social roles of ‘prisoner’ and ‘patient’ have been documented^17^ and were also present in our study as the professional groups used a range of terms including ‘*prisoner*’, ‘*patient*’ ‘*prisoner/patient*’, and ‘*the men and women in our care*’. Tensions between care and custody have been found in other studies on prison healthcare,^5^^,^^21^ but this is the first to explore these specifically in cancer care and to highlight that prison officers struggle to reconcile these roles. We found that control measures and limited opportunities for choice in prison healthcare impacted patients' experience of cancer care in terms of symptom management, accessing information about their condition and family involvement in their care. Not all barriers are specific to patients in prison, some (i.e., prolonged diagnosis) are experienced by people residing in the community but they are exacerbated by the prison environment. Our study adds to existing knowledge by offering an in-depth multi-perspective, inclusive of the prisoner-patient voice that demonstrates the complexity of cancer care in custodial settings.

Due to COVID-19 we had to stop our fieldwork early, nevertheless the 55 interviews conducted provided detailed information from a broad range of participants. One-off interviews offer a snapshot of the lives of people although follow-up interviews and observations could have offered even more insight into the lived experience of people with cancer in prison and those who care for them. However, as the first study exploring this issue, our interviews already offer ample evidence on how cancer care was experienced and potentially could be improved.

This is the first time that NCPES data have been analysed for patients in prison and we were not able to match patients’ NCPES response on tumour site or stage, which may confound our findings. We also did not have sufficient data to address potential confounding by ethnicity. Potentially, the analysis is biased due to data sparsity.^22^ In addition, we do not know how representative the patients responding were of all those diagnosed in prison. It is possible that they were not representative as we know that patients from lower socioeconomic groups or with other ethnic backgrounds than White are less likely to respond to surveys and that lower literacy in the prison population would likely decrease response rates. Although this appeared to be mostly similar for responders from prison and the general population, NCPES responders did not answer all the survey questions, or the questions did not apply to their circumstances further limiting the strength of the findings. Nevertheless, the NCPES results appear to support the findings from the qualitative interviews.

We identified several barriers and enablers to getting a diagnosis and treatment. Whilst oncologists might treat people in prison ‘the same’, this sameness can unintentionally lead to further disadvantage if the constraints of the prison environment are not understood. We also found that each prison adopts different ways of caring for their population. National guidance and the sharing of best practice could potentially improve cancer care for this population. Better means of communication within and between institutions is a further way in which cancer care and specifically the patient experience could progress. Our results are similar to other studies on healthcare in prison, showing that there is awareness of the disadvantages and structural barriers to care for those residing in prison.^5^^,^^17^^,^^21^ With a growing and ageing prison population there is an increasing need for these barriers to be addressed in both policy and practice to enable imprisoned cancer patients to access equivalent care to that in the community.

## Contributors

JA, ED and ML designed the study and decided the analytic approach for the qualitative phase of the study although all authors were involved in conceiving the study as a whole. JA, RV, SW, AX & SW conducted the interviews and analysed the qualitative data. JA and RV accessed the data. JA verified the data. JH and ML accessed the NCPES data and conducted the statistical analysis. ML verified the data. RV and JA developed the initial draft of the manuscript. All authors critically revised the manuscript and approved the final manuscript. JA, ML and ED had final responsibility for the decision to submit the manuscript.

## Data sharing statement

NCPES data for this study is collated, owned, maintained and quality assured by the National Cancer Registration and Analysis Service within NHS England. The authors do not own these data, and therefore are not permitted to share or provide this data other than in scientific communication format. We do not have participant consent to share the qualitative data and we are not able to share or provide this data other than in scientific communication format.

## Declaration of interests

The authors declare that they have no conflict of interest.

## Appendix A Supplementary data

Armes cancer in Prison Supplemntary File 1

Armes cancer in Prison Supplemntary File 2
