# Supplementary material for: Cancer in prison: barriers and enablers to diagnosis and treatment
Source: eClinicalMedicine. 2024 Apr 29;72:102540. doi: 10.1016/j.eclinm.2024.102540 (PMC11247143; doi:10.1016/j.eclinm.2024.102540)
Supplement: Armes cancer in Prison Supplemntary File 1 [file mmc1.docx]

**Supplementary File 1. National cancer patient experience survey responses, persons with a cancer diagnosed in prison compared with those diagnosed in the general population.**

|  |  | General population | | Prison | |  |
| --- | --- | --- | --- | --- | --- | --- |
| Question # | Preferred answer | Number | Proportion | Number | Proportion | P value* |
|  |  | 390 |  | 78 |  |  |
| 1 | No | 89 | 23% | 29 | 37% |  |
|  | Yes | 222 | 57% | 26 | 33% | <0.0001 |
|  | Missing | 79 | 20% | 23 | 29% | 0.071 |
| 3 | No | 73 | 19% | 29 | 37% |  |
|  | Yes | 286 | 73% | 39 | 50% | <0.0001 |
|  | Missing | 31 | 8% | 10 | 13% | 0.165 |
| 11 | No | 90 | 23% | 42 | 54% |  |
|  | Yes | 235 | 60% | 20 | 26% | <0.001 |
|  | Missing | 65 | 17% | 16 | 21% | 0.412 |
| 14 | No | 90 | 23% | 26 | 33% |  |
|  | Yes | 235 | 60% | 45 | 58% | 0.134 |
|  | Missing | 65 | 17% | 7 | 9% | 0.086 |
| 17 | No | 86 | 22% | 19 | 24% |  |
|  | Yes | 280 | 72% | 56 | 72% | 0.734 |
|  | Missing | 24 | 6% | 3 | 4% | 0.425 |
| 18 | No | 17 | 4% | 11 | 14% |  |
|  | Yes | 130 | 33% | 20 | 26% | 0.001 |
|  | Missing | 243 | 62% | 47 | 60% | 0.733 |
| 26 | No | 40 | 10% | 14 | 18% |  |
|  | Yes | 194 | 50% | 37 | 47% | 0.087 |
|  | Missing | 156 | 40% | 27 | 35% | 0.374 |
| 48 | No | 37 | 9% | 21 | 27% |  |
|  | Yes | 226 | 58% | 28 | 36% | <0.0001 |
|  | Missing | 127 | 33% | 29 | 37% | 0.430 |
| 54 | No | 14 | 4% | 15 | 19% |  |
|  | Yes | 245 | 63% | 31 | 40% | <0.001 |
|  | Missing | 131 | 34% | 32 | 41% | 0.208 |
| 55 | No | 105 | 27% | 22 | 28% |  |
|  | Yes | 180 | 46% | 17 | 22% | 0.019 |
|  | Missing | 105 | 27% | 39 | 50% | <0.0001 |
| 63 | No | 17 | 4% | 13 | 17% |  |
|  | Yes | 300 | 77% | 46 | 59% | <0.0001 |
|  | Missing | 73 | 19% | 19 | 24% | 0.252 |
| 64 | No | 89 | 23% | 32 | 41% |  |
|  | Yes | 178 | 46% | 30 | 38% | 0.007 |
|  | Missing | 123 | 32% | 16 | 21% | 0.052 |
| *P-value for chi square test yes vs. no and any answer vs. missing. Missing answers include answers indicating that the question was not applicable.* | | | | | | |
