# Supplementary material for: Cancer in prison: barriers and enablers to diagnosis and treatment
Source: eClinicalMedicine. 2024 Apr 29;72:102540. doi: 10.1016/j.eclinm.2024.102540 (PMC11247143; doi:10.1016/j.eclinm.2024.102540)
Supplement: Armes cancer in Prison Supplemntary File 2 [file mmc2.docx]

**Supplementary File 2: Participant quotations**

| **Patients** | **Oncology specialist** | **Custodial staff** | **Prison healthcare** |
| --- | --- | --- | --- |
| **COMMUNICATION** | | | |
| **Between patients & GPs** | | | |
| **Getting a referral**  “You’ve got to write out paper apps to see a doctor. That can take time, so you’ve got all that, playing up there, thinking, “Oh God, I need to get this sorted out straight away.” But you can’t because it’s out of your hands; there’s nothing you can do until you get that appointment with the doctor” (Man, 50s, haem cancer)  “But if I ever have something I’m not too sure about, I’ll just write, “I’ve got a pain here, but due to my [cancer] history …” and they’re going to need to look at that, you do get looked at a lot quicker (Man, 80s, multiple cancers)  “Every day I was telling them, and they just kept telling me that they’d got booked appointments. Then they [healthcare] told me “Oh, you’ve got a cold, it’s a gland,” and then it wasn’t a gland, it was a thyroid. “We’re booking him in to have it operated on, it’s just your thyroid.” But it turns out it wasn’t a thyroid, it was cancer” (Man, 20s, haem cancer).  “It took around five months for the doctor to send me to hospital to get diagnosed. I’d never been ill up until this really, like normal illness and stuff but nothing serious. And I was going to the doctor a lot with just being extremely tired” (Man, 20s, haem cancer)  **Post-prison move**  “I was first diagnosed when I was in [different prison], with all the blood tests they do when you go into prison. And then I was referred to [local hospital] and seen a consultant there, and I was just diagnosed with CLL.” (Man, 50s, haem cancer) | **Post-prison move**  “We are quite often asked to see people who have been transferred to a prison that is local to us who are not a local resident. They might be in one prison, then moved to a different prison and there is a complete lack of continuity of care (…) We were the third specialist bladder cancer team that were meeting this patient within 6 months.” (Surgeon) |  | **Getting a referral**  “It could be a patient who self-presents, who puts an app in, ‘I've found a lump’, otherwise we wouldn’t know, and that gets the ball rolling with the initial investigations, scans. Or it could be an incidental finding, so they’ve come for something else but then through either doing blood sampling or a thorough examination they’ve found something else” (Lead Nurse)  **Malingering**  “The other bit that happens is malingering. Malingering happens; people tell you they are bleeding, say bleeding in the urine and bleeding in the rectum and things like that, and then you find out that the episode happened to be the day before their court case.” (GP)  **Mental health & substance abuse**  “To be honest, a minority of them are people who have got physical health diseases. The majority of them is medication review, mental health review, but the most difficult or challenging consultations are those around substance misuse and the issuing of prescriptions which are potentially abusable within the prison service” (GP)  “I mean, as much you build up relationships with prisoners because you see the same ones in and out quite a lot, it’s not the same thing. Unfortunately, a lot of prisoners are not interested in their health, they are interested in other things. And the problem is that by the time they are interested in their health, they have got serious problems and an awful lot of time and money in prisons is dedicated to their substance misuse problems and their mental health problems.” (GP) |
| **Between patients and the oncology team** | | | |
| “I tried to get in touch with some organisations, but that was so difficult because I think I was the first person. Like Macmillan. By the time they’d allowed me to have the freephone number and an extended amount of time on the telephone so that you can wait to get through to all the right channels, then I was actually over all the treatment” (Woman 60s, breast cancer) | “All patients are given a phone number to ring if they are in that situation [of developing a fever or infection]. That is clearly not straightforward if you are on your own in prison” (Urologist)  “The problem was more that once we finished the consultation, and then if we had to follow it up with a phone call, how we are doing, that was not possible” (Medical Oncologist) |  |  |
| **Between professional groups** | | | |
|  | “it is just really hard communicating when the prison healthcare system seems pretty thin on the ground and stretched. It’s difficult to get hold of people to discuss and to try and make a plan” (Clinical Oncologist)  “it’s a bit more of a logistics one just to try to keep on top of the communication with the caseworkers at the prison, to make sure they’re aware of our rules and we’re aware of their rules, and they’re aware of when the patient needs to attend and what they’re coming for.” (Chemotherapy Scheduler)  “A lot of the discussions we have with the prison is, ‘*What are you able to deliver?*’ The problem is he has to be in a prison because he is in detention, but then what is the situation when they can’t deliver? Does he end up staying in hospital, which also isn’t the best place? With this particular case, that was some of the challenges. (Specialist Registrar)  “I think his contact with us and his contact as an inpatient when things were not as good was probably prolonged because of where he was going to have to go back to when he was discharged from hospital.” (Specialist Registrar) | **Medical Confidentiality**  “I believe you do get a bit of a brick wall sometimes [from prison healthcare] and you’re not getting the full picture. Or things are delayed from the outside hospital with information, and I get to hear the information from the prisoner before it comes from the hospital to the prison.” (Prison Officer)  “But we wouldn’t have been sat down and been told about the diagnosis with them, it would just filter through to us, and then we would start putting things into place for them” (Prison Officer)  “I’ve almost got to go and get a piece of paper signed each time for them to information share. Sometimes, if we had the full picture at the beginning, it would help me with my position, especially if there is going to be that risk of that person falling into that palliative care. If I’m prepared for it, then I’m prepared to be able to support them in a better way” (Prison Officer) | **Medical Confidentiality**  “I think sometimes the nurses in the hospital can be quite difficult to communicate with us. They’ve very reluctant to communicate with us, even when we’ve had to set passwords up and things like that for them to communicate with us, they will only give us the bare information, the minimum” (Nurse)  “Some of the NHS are really funny about it, they won’t send stuff to [Prison healthcare email] but if you’ve got an NHS [email], they’ll email you to an NHS” (Senior Staff Nurse)  “I just send a letter to the officers on the wing to say, “Look, he’s on this treatment, these are the side effects. Please be aware. These are the phone numbers to call should he have anything.” So, they keep that in their office, so they’ve got all that” (Clinical Nurse Specialist)  “A lot of the time they [patients] won’t have anybody else with them. So if they’re being bombarded with information, we [healthcare] don’t know what information has been given because obviously the prison officers don’t come back and tell us” (Clinical Nurse Practitioner) |
| **CONTROL AND CHOICE** | | | |
| **Preparation for appointments** | | | |
| “No, I know when I’m going. I’m not meant to, do you know what I mean? It’s every 21 days they have to do it” (Man 20s, head & neck cancer)  “I didn’t know I was going. I hadn’t written my questions down. I keep asking odd questions [in the hospital].” (Man 80s, head & neck cancer)  “Quite simply, my operation is at 7:30, I have to be in [Hospital], so I've got to leave here at 6:30. They require me to have washed my hair before I go. It’s very difficult for prisoners to wash their hair in the prison. I need to do it the night before, but will I be told that I'm going to [Hospital] the following morning? Probably not” (Man, 70s, multiple cancers) | “I don’t know this for 100 per cent sure, but I think when booking appointments the bookings team liaise with the prison. They don’t tell the prisoner, to try and de-risk anything from that point of view” (Specialist Registrar)  *Information giving to patients*  “I think information-giving is really difficult because you always feel slightly constrained, you are saying to them, “We will give you chemotherapy every three weeks.” And you are thinking, “Can I say that?” Because they are not supposed to know when they are coming (Clinical Nurse) | “They are not allowed to know when they are going, but they’ll have an idea when they’re going. We’ll take the gentleman for his chemotherapy appointment, he’ll have his chemo, he’ll come away. The hospital will then write to the prison with the new date for the next session. And he won’t know when he’s having the next one” (Prison Officer) | “Well usually patients can’t know when they’re going out to hospital because there could be a security breach. So there’s the element of surprise when they may or may not know they’re going and that morning they get up and have to go to hospital. If they need to be nil by mouth they may know the night before, but that’s rare” (Head of Healthcare) |
| **Treatment** | | | |
| **Chemotherapy**  “I have three lots of chemo; every 21 days I have to go and have it done. It’s only once every 21 days, but I’m on three different types. I don’t know what they’re called,” (Man 20s, head & neck cancer)  **Radiotherapy**  “Well, it is cancer because I am having radiotherapy, possibly some time this month. I don’t know when because they don’t tell you.” (Man 80s, lung cancer)  **Hormone therapy**  “I’m on tablets now. They’ve not said anything about any further treatment or whatever, or anything. I don’t think they tell you if they’ve received anything from the specialist; they only tell you what they want you to know.” (Man 70s, urological cancer)  **Surgery**  “All I had was operations, operations, operations” (Man 50s, head & neck cancer) | **Equivalent treatment plans**  “The patient, depending on their diagnosis, stage, things like that, would be offered the best treatment for them. Any cancer patient, regardless of whether they are a prisoner or not, will be discussed in a multidisciplinary team meeting – a group of professionals who then come up with what they think is the best treatment option for that patient.” (Radiographer)  “Cancer is very much a standardised treatment based on evidence so we try to do all we can. I think in terms of his treatment, it wasn’t any different to what it would have been.” (Specialist Registrar) |  | **Communicating about treatment**  “So, we’ve got a guy who’s on chemotherapy tablets in the prison at the moment, so I just send a letter to the officers on the wing to say, “*Look, he’s on this treatment, these are the side effects. Please be aware. These are the phone numbers to call should he have anything*.” So, they keep that in their office so they’ve got all that.” (Clinical Nurse Specialist)  “So usually if they are going to start active treatments, we liaise with wherever they're going to if they were going to have a course of chemotherapy, we’ll get all those dates prior to it and then admin will clear them all with security, because they're only allowed so many patients out a day, we triage that list so that patient then becomes priority. So if there's other people on the list that day, they’ll be taken off and moved elsewhere. So we plan ahead so it doesn’t affect their chemotherapy or radiotherapy or whatever treatment they're having” (Lead Nurse) |
| **Managing treatment side-effects** | | | |
| “I have suffered very severe side effects from the radiotherapy. This is not necessarily relevant to having that treatment from prison, but it’s difficult to have side effect management in prison. All night sweating you can cope with, but anal bleeding is not easy to handle, and I have that every day.” (Man 70s, multiple cancers)  “Obviously, they’ve got to kill my immune system and then try and rebuild it. In here, you’re just open to infections. Plus, staff come in with coughs and colds, and it’s just a dirty environment.” (Man, 20s, haem cancer)  “I’ve not had any side effects at all. But no, some lads on the wing do have side effects, and sometimes they’ll go, “I’m not going to air today, I don’t feel too well,” or this sort of stuff.” (Man, 80s, multiple cancers) | “He had two episodes of febrile neutropenia and actually he felt that his first one was ignored (…) So actually getting him to hospital as an emergency was probably quite difficult and it must have been difficult for them [the prison] to facilitate.” (Clinical Nurse Specialist)  “I don’t think that [being in prison] would necessarily change my approach to explaining side-effects to them, because my assumption would be that, if they have a prison doctor and the prison doctor is completely aware of a patient and the management plan that they’re undergoing” (Clinical Oncologist)  “You have to explain everything because he still needs to be treated as a normal patient. If you have any new symptoms, if you have any new worries, if you have any new concerns, you ring the hotline. Then again it is back on the prison. ‘*He has rung and we want to see him. You have got to arrange for him to come up*.’ If a person rang me from [place], say, and they were ill, I would tell them to go to A&E” (Specialist Registrar) | “I believe if someone was given anything like that [chemotherapy], healthcare would generally come and give us some sort of handover, saying, “*This person’s having this therapy, and these are going to be the side effects*” (Prison Officer)  “One [patient] had a brain tumour (…) he was going up to hospital either every day or every couple of days for treatment (…). But it was *the actual prisoner who lived with him* who came and told me if he was struggling. I don’t think staff are fully aware of what to look out for” (Prison Officer) | “We've had other patients where we’ve been managing their side effects from their cancer treatment, or explaining their diagnosis to them because they don’t really understand it because they don’t take it in when it’s told to them in hospital, or because they can't read very well – that kind of thing” (Clinical Nurse Specialist)  “There’s a protocol in place, around chemotherapy and around blood transfusions as well, with escalation plans in place if someone’s bloods are indicating a neutropenia or something like that, what to do. That’s shared with both prison staff and with the clinical staff so they know exactly what the contingency plan is, if XYZ happens” (Head of Healthcare) |
| **Managing emotions** | | | |
| “I’ll have the chemo, and as the days go on, you feel bad for the first few days, then you start feeling a bit better again. But it’s your emotions; it plays with your head, badly. I just have to keep telling myself I’m going to get better, but it does play with your emotions. I had it going last Monday, and then Monday night they didn’t give me my medication that they’re meant to give me, and I just flipped. I knew when I was doing it that I was just doing it because I was kicking off for no reason, but I couldn’t control it; your head just goes.” (Man, 20s, haem cancer)  “I’ve been told that I’ve got a really aggressive cancer, and I’m not being given any time to process it.” I’m a man, I want to go and cry in my cell on my own, just have a night where I try and get my head around everything.” (Man, 20s, haem cancer)  “When you’re vulnerable or unwell, or mentally vulnerable, the officers will all decide, they’ll have a little sit down and a chat with you and work out things that they can do to help you, people you can see, and how many times they’d like to check on you so that you’re still alive. So, they did that, and I didn’t really need that.” (Woman, 60s, breast cancer) |  |  |  |
| **CARE AND CUSTODY** | | | |
| **Getting to hospital** | | | |
| **Missing appointments**  “The main thing I think that sometimes breaks down is getting people to the hospital when they need to be. That’s the only thing where mine went wrong” (Man, 60s, urological cancer)  **Missing scans**  “I’ve never missed a chemo appointment. The one [doctor] was really cross about was the scan. I was supposed to have had one before the last appointment and I haven't and she said, “Why not?” and I said, “I don't know”. That’s like blaming the prisoner for not having a key” (Woman, 40s, breast cancer) | **Missing appointments**  “They don’t turn up to an appointment and we end up trying to chase it up, phone the original prison, and they say ‘No, they're not there.’ I've spent hours trying to track people down in the past, not being willing to tell me where somebody is” (Clinical Oncologist)  **Escorts - arranging appointments**  “It still has been quite hard work, negotiating with the prison and actually getting him into appointments. I've gone to the trouble of trying to liaise with our x-ray department for him to have the ultrasound scan on the same day he comes to see me, to help the prison out so that he’s only [needed] one trip under escort out” (Clinical Oncologist) | **Missing appointments**  “Unless something has gone catastrophically wrong, they will never miss their appointment. And obviously because we know they’re going off for chemo, we know how important that is. So no, they should never, in an ideal world, miss their appointments” (Prison Officer)  **Hospital escorts**  “It’s about making sure that a diagnosis is reached quickly, and that’s about us communicating to the medical staff, the medical staff communicating to the relevant Oncology department, making sure that we’ve got staff available to escort” (Custodial Manager) | **Hospital escorts**  “Now, the challenge with that is, the prison has capacity issues and resource issues about taking anybody out for any healthcare appointment. So, the prison currently allows 3 outside appointments in a day, which means that you are often the people who pick and choose who goes out.” (Prison Healthcare) |
| **Handcuffing**  “It’s a bit of a thing at the minute, because I’m 80 years old, when you go for the scan, they lock the door, nobody can go in anyway, but they still insist on being cuffed.” (Man, 80s, multiple cancers)  “[when going for] a scan they have to take your handcuffs off and put plastics on (…) They’re plastic handcuffs, and they haven't got anything to open them with they have to use this prehistoric tool to open it.” (Man 50s, GI cancer)  “I don’t get handcuffed when I go to the hospital. There is a list of a few of us that don’t; we just go down with two prison officers.” (Man 70s, urological cancer) | **Handcuffing**  “But yes, typically, they are handcuffed when we see them. Obviously if you need to examine the patient, often there is some arrangement to allow that. But it’s not ideal” (Clinical Oncologist)  “It is so obvious when they [patients from prison] walk into the department handcuffed to two burly [prison officers] … Everyone looks then. How can we try and reduce some of the stigma attached to that?” (Radiographer) | **Handcuffing**  “[I]f they are cuffed, we are sensitive to that and we don’t parade them round and bark orders at them in the hospital. We’re sensitive to their needs and the public needs, because we’re the face of the prison service when we’re out there” (Custodial Manager)  **Public Safety**  “But we’ve also got to be aware that these people are a risk to the public, so we’ve got our job to do there in protecting the public. And we try to just blend in as much as two prison officers and a prisoner handcuffed together can blend in” (Prison Officer) | **Handcuffing**  “They obviously will go out in handcuffs with two officers.” (Head of Healthcare)  “And I think 98% of our cancer patients are not cuffed when they go to their appointments either. I’ve got a list of 36 patients that are not cuffed” (Clinical Nurse Specialist) |
| **Prison Officers in consultations** | | | |
| **Presence Prison officers**  “The chance to sit and talk to a specialist without somebody else in the room [would be great]. While they [the officers] might be sympathetic, it’s not their treatment. Because there are questions I would like to ask my consultant but I don't feel comfortable” (Woman, 40s, breast cancer)  “You have to have an enema every time you have radiotherapy and having an enema with prison escorting officers is difficult. There were different escorting officers each time” (Man, 70s, urological cancer) | **Presence prison officers** “It is always challenging, and you always ask permission from the patient [for the prison officers to be present]. I don’t understand the ins and outs of the law. I presume that whoever is in charge of bringing him has to be there.” (Specialist Registrar)  “She [a patient] was a young woman who was really, really frightened about losing her fertility. So there was a lot of discussion about that and that was all done in the presence of strangers” (Clinical Nurse Specialist)  *Ignoring presence prison officers*  “We try to blank it out [the presence of prison officers], so when you have a patient in front of you, whether you have a prison officer or whether they are handcuffed or not handcuffed, you try to just have an absolutely normal conversation as much as you possibly can” (Medical Oncologist) | **Presence Prison officers**  “I’ve also been on escorts where they’ve gone out for their treatment, their chemotherapy. And obviously you’re on a ward with other people who are suffering from cancer and having treatment and you’re there with a closeting chain sometimes. It’s not great.” (Prison Officer)  *Trying to absorb information*  “We’ll go, we’ll try and absorb some of the information to him, just in case he misses something, so we can tell him “They said that and said this,” just try and make it as comfortable as we can for them” (Prison Officer)  *Zoning out during consults*  So obviously we know we’re hearing stuff that is for him. Me, personally, when stuff goes on between him and his doctor, I try not to listen unless the doctor will ask questions like, at the prison is this available to them.” (Custodial Manager) |  |
